# Molecular testing for adolescent and young adult central nervous system tumors: A Canadian guideline

**DOI:** 10.3389/fonc.2022.960509

**Published:** 2022-09-23

**Authors:** Mary Jane Lim-Fat, Maria Macdonald, Sarah Lapointe, Seth Andrew Climans, Chantel Cacciotti, Manik Chahal, Sebastien Perreault, Derek S. Tsang, Andrew Gao, Stephen Yip, Julia Keith, Julie Bennett, Vijay Ramaswamy, Jay Detsky, Uri Tabori, Sunit Das, Cynthia Hawkins

**Affiliations:** ^1^ Division of Neurology, Department of Medicine, Sunnybrook Health Sciences Centre, University of Toronto, Toronto, ON, Canada; ^2^ Department of Oncology, London Health Sciences Centre, Schulich School of Medicine and Dentistry, University of Western Ontario, London, ON, Canada; ^3^ Division of Neurology, Department of Medicine, Centre Hospitalier de l'Universite de Montreal, Montreal, QC, Canada; ^4^ Department of Paediatrics, Division of Pediatric Hematology/Oncology, London Health Sciences Centre, London, ON, Canada; ^5^ Department of Medical Oncology, BC Cancer Vancouver Centre, Vancouver, BC, Canada; ^6^ Department of Pediatrics, Division of Child Neurology, CHU Sainte-Justine, Montreal, QC, Canada; ^7^ Radiation Medicine Program, Princess Margaret Cancer Centre, University Health Network, Toronto, ON, Canada; ^8^ Department of Laboratory Medicine and Pathobiology, University Health Network, University of Toronto, Toronto, ON, Canada; ^9^ Department of Pathology & Laboratory Medicine, Faculty of Medicine, University of British Columbia, BC, Canada; ^10^ Department of Laboratory Medicine and Pathobiology, Sunnybrook Health Sciences Center, University of Toronto, Toronto, ON, Canada; ^11^ Division of Haematology/Oncology, The Hospital for Sick Children, Toronto ON, Canada; ^12^ Department of Radiation Oncology, Sunnybrook Health Sciences Center, University of Toronto, Toronto, ON, Canada; ^13^ Division of Neurosurgery, Department of Surgery, University of Toronto, Toronto, ON, Canada; ^14^ Department of Paediatric Laboratory Medicine, The Hospital for Sick Children, Toronto ON, Canada

**Keywords:** AYA, precision oncology, CNS tumor classification, molecular testing, targeted therapy

## Abstract

The 2021 World Health Organization (WHO) classification of CNS tumors incorporates molecular signatures with histology and has highlighted differences across pediatric vs adult-type CNS tumors. However, adolescent and young adults (AYA; aged 15–39), can suffer from tumors across this spectrum and is a recognized orphan population that requires multidisciplinary, specialized care, and often through a transition phase. To advocate for a uniform testing strategy in AYAs, pediatric and adult specialists from neuro-oncology, radiation oncology, neuropathology, and neurosurgery helped develop this review and testing framework through the Canadian AYA Neuro-Oncology Consortium. We propose a comprehensive approach to molecular testing in this unique population, based on the recent tumor classification and within the clinical framework of the provincial health care systems in Canada.

**Contributions to the field:**

While there are guidelines for testing in adult and pediatric CNS tumor populations, there is no consensus testing for AYA patients whose care occur in both pediatric and adult hospitals. Our review of the literature and guideline adopts a resource-effective and clinically-oriented approach to improve diagnosis and prognostication of brain tumors in the AYA population, as part of a nation-wide initiative to improve care for AYA patients.

## Introduction

Advances in molecular techniques and concerted efforts to characterize signatures at the genomic, epigenomic, transcriptomic and proteomic level have provided a deeper understanding and appreciation of the biology, molecular classification, and behavior of brain tumors. The 2016 revision to the WHO classification formally introduced the integration of molecular diagnostics to complement histological diagnosis and grading (1). The classification was further detailed and updated by the Consortium to Inform Molecular and Practical Approaches to CNS Tumor Taxonomy (cIMPACT-NOW), which has now published seven updates (2–8). The 2021 WHO classification (5th Edition) (WHO CNS5) reflects these updates and incorporates molecular signatures with histology for an integrated diagnosis of brain tumors (9).

The adolescent and young adult (AYA) population is defined by the National Cancer Institute as patients aged 15–39. Primary brain tumors remain a major cause of morbidity and mortality, and AYA patients represent 30% of the population with CNS cancers (10). This population is understudied across the cancer spectrum and represents an age group where transition from pediatric to adult care can represent an important barrier to specialized care and to clinical trials (11). AYA CNS tumors span a number of different subtypes including glioma (both adult and pediatric types), medulloblastoma/embryonal tumors and ependymoma. Between 2013–2017 in Canada, 250 AYA patients were diagnosed annually with glioma, 38 were diagnosed with glioneuronal tumors, and 13 with embryonal tumors (12), although this may be an underestimate due to heterogeneous testing strategies. Currently, there are no defined consensus diagnostic or treatment guidelines for patients in this age group. However, there is a clear need for multidisciplinary care involving both pediatric and adult neuro-oncologists, neurosurgeons, radiation oncologists, and neuropathologists as Canadian patients with “pediatric-type” tumors are often treated at adult medical centers and vice-versa.

While WHO CNS5 presents recommendations for molecular testing across age groups, some markers are more clinically relevant in the AYA context, and practical implementation nationally will need to occur within the limitations of a single-payer provincially reimbursed system. In Canada, testing for molecular markers in brain tumors remains practitioner- and institution-dependent, although recommendations exist across some provinces, which are mandated by each provincial governing body (e.g., Cancer Care Ontario, BC Cancer, etc).

The purpose of this publication is to inform and engage Canadian neuro-oncologists, radiation oncologists, neuropathologists and neurosurgeons regarding the molecular work-up that is clinically relevant for the care of AYA CNS patients based on the 2021 WHO classification (5th Edition) (9). Here, we present consensus guidelines for molecular testing in the AYA CNS population, based on review of the literature and expert opinion, focusing on clinical care in the context of diagnosis, treatment selection and prognostication.

## Glioma

The WHO CNS5 now classifies gliomas, glioneuronal tumors and neuronal tumors into six different families: adult-type diffuse gliomas, pediatric-type diffuse low-grade gliomas, pediatric-type diffuse high-grade gliomas, circumscribed astrocytic gliomas, glioneuronal and neuronal tumors, and ependymal tumors (9). Gliomas (of all types) account for approximately 43% of all brain and other CNS tumors in AYAs, and about 83% of all malignant tumors (13). Despite their monikers, pediatric-type tumors sometimes occur in young adults and adult-type tumors may occur in adolescents. Still, the exact age-stratified incidence using the new classification system remains undetermined and is an active area of research. This new recognition of the clinical and molecular distinctions between diffuse gliomas that primarily occur in adults (adult-type gliomas) and those that primarily occur in children (pediatric-type gliomas) is likely the most important change in the WHO CNS5. This fundamental distinction aims to better the understanding of pathobiology and prognosis, improve care, and promises to inform more biologically sensible patient enrollment into clinical trials. Thus, the information presented below will also be stratified by adult- vs pediatric-type gliomas (**Table 1**).

**Table 1:** Recommended biomarkers for testing and their clinical implications in AYA glioma.

	Genes/Molecular profiles characteristically altered	Clinically relevant biomarkers (diagnostic, predictive or prognostic)
*Adult-type diffuse gliomas*		
Astrocytoma, IDH-mutant (CNS WHO grade 2, 3, 4)	IDH1, IDH2, ATRX, TP53, CDKN2A/B	ATRX nuclear loss is diagnostic for astrocytic- lineage tumors in an IDH-mutant glioma TP53 mutations are commonly found in astrocytomas CDKN2A/B homozygous deletion is a marker of poor prognosis and upgrades Grade 2/3 IDH-mutant astrocytomas to Grade 4 astrocytomas
Oligodendroglioma, IDH-mutant, and 1p/19q-codeleted (CNS WHO grades 2, 3)	IDH1, IDH2, 1p/19q, TERT promoter, CIC, FUBP1, NOTCH1	1p/19q codeletion distinguishes oligodendroglioma from astrocytoma, within IDH-mutant glioma
Glioblastoma, IDH-wildtype (CNS WHO grade 4)	IDH-wildtype, TERT promoter, chromosomes 7+/10-, EGFR	IDH-wildtype, and one of: TERT promoter mutation; chromosome 7+/10-; or EGFR amplification, defines molecular GBM irrespective of histologic grade MGMT promoter methylation is a prognostic biomarker independent of treatment with alkylating chemotherapy and a predictive biomarker of benefit from alkylating chemotherapy in patients with IDH-wildtype glioblastoma
*Pediatric-type diffuse gliomas*		
Low-grade glioma (IDH-wildtype)	BRAF, FGFR1, FGFR2, MYBL1, MYB, or other MAPK alterations, CDKN2A	In pediatric LGG, homozygous deletion in CDKN2A carry a worse prognosis. RAF/RAS/MAPK alterations can offer targeted therapy options
High-grade glioma (hemispheric) High-grade glioma (midline)	H3 G34R H3 K27M	In pediatric HGG, H3 G34R and H3 K27M alterations are diagnostic and confer a poor prognosis
High-grade glioma (IDH-wildtype and H3-wildtype)	BRAF V600E*, FGFR1*, MYBL*, MYB*, MYCN, PDGFRA, EGFR, p53, or other MAPK alterations, MLH1, MSH2, MSH6 and PMS	*Mutations in BRAF V600E, FGFR1, MYBL, MYB carry a better prognosis and are more common in low-grade glioma RAF/RAS/MAPK alterations can offer targeted therapy options
*Glioneuronal tumors*		
Dysembryoplastic neuroepithelial tumor, Ganglioglioma, Multinodular and vacuolating neuronal tumor, and others	FGFR1 in DNETs, MAPK alterations in MVNTs	RAF/RAS/MAPK alterations can offer targeted therapy options

### Adult-type gliomas

In the WHO CNS5, neoplasms are now graded within rather than across different tumor types (1). This mainly impacts the common diffuse gliomas of adults, which were previously divided into 15 entities but now include only three: astrocytoma, isocitrate dehydrogenase (IDH)-mutant; oligodendroglioma, IDH-mutant and 1p/19q codeleted; and glioblastoma, IDH-wildtype. The loss of nuclear ATRX chromatin remodeler (ATRX) in an IDH-mutant glioma is now sufficient for the diagnosis of an astrocytic lineage tumor without the need for 1p/19q codeletion analysis. Conversely, 1p/19q codeletion status should be determined in all IDH-mutant gliomas with retained nuclear expression of ATRX (14).

In the new classification, molecular parameters contribute to tumor grade. All IDH-mutant diffuse astrocytic tumors are now considered a single type—astrocytoma, IDH-mutant—and are graded as 2, 3 or 4 (with “anaplastic” no longer used). The presence of cyclin-dependent kinase inhibitor (CDKN) 2A/B homozygous deletion, even in the absence of microvascular proliferation or necrosis, portends a worse prognosis and therefore is classified as astrocytoma, IDH-mutant, CNS WHO grade 4. In the WHO CNS5, the term glioblastoma (GBM) is no longer used to refer to IDH-mutant astrocytic gliomas. GBM is now defined as a diffuse astrocytic glioma with neither mutations in the *IDH* or histone *H3* genes, and is characterized by microvascular proliferation and/or necrosis, and/or one or more of three genetic parameters: telomerase reverse transcriptase (TERT) promoter mutation, epidermal growth factor receptor (EGFR) amplification, or combined gain of entire chromosome 7 and loss of entire chromosome 10 (1). However, caution needs to be exercised, particularly in diffuse astrocytomas (Grade 2) with isolated TERT promoter mutations (15, 16). The majority of GBM, especially those with classical histological features, are diagnosed in the elderly population. In IDH-wildtype diffuse astrocytomas without any of these three genetic alterations, especially among AYA patients, pediatric-type gliomas must be considered (see below) (1). Subtypes such as gliosarcoma and giant cell GBM are no longer listed in WHO CNS5 (1).

### Pediatric-type gliomas

#### Pediatric-type diffuse low-grade gliomas

Under WHO CNS5, pediatric-type low-grade gliomas are subdivided into *MYB*- or *MYBL1*- (from the avian **my**elo**b**alstosis viral oncogene) altered diffuse gliomas, mitogen-activated protein kinase (MAPK)-altered diffuse gliomas (commonly BRAF or FGFR1 alterations), angiocentric glioma (MYB::QKI fusion), or polymorphous low-grade neuroepithelial tumor of the young (FGFR2 fusions) (9). This is particularly important to recognize in the AYA population, where gliomas with alterations in the MAPK pathway or *MYB* and *MYBL1* have distinctly different prognoses from other glioma subtypes and offer possibilities for targeted therapy (5). From a histopathological standpoint, pediatric- type LGGs with alterations in the MAPK pathway are diffuse gliomas with low density of cells with mild atypia. They typically have diffuse immunopositivity to OLIG2 and variable expression of GFAP. *MYB* or *MYBL1-*altered tumors on the other hand consist of relatively monomorphic cells of glial origin with bland round to spindled nuclei within a fibrillar matrix and may have vague angiocentric polarity. They are typically immunoreactive for GFAP but negative for OLIG2.

#### Pediatric-type diffuse high-grade gliomas

In contrast to adult-type gliomas (which may transform from low-grade tumors), pediatric-type HGGs arise from distinct molecular drivers (17). Within the AYA group, pediatric-type diffuse high-grade gliomas (HGGs) can be further subdivided into 1) diffuse glioma, H3 K27-altered; 2) diffuse hemispheric glioma, H3 G34-mutant; and 3) diffuse pediatric type HGG, H3-wildtype and IDH-wildtype.

Diffuse gliomas with amino acid substitutions in lysine 27 of H3.3 or H3.1 of (*H3-3A* or *H3C2* genes, respectively) are typically midline tumors (brain stem, thalamus or spinal cord) that occur in children and younger adults. Microscopically, they are usually astrocytic but can also show varied cytology with piloid, oligodendroglial, giant cell, epithelioid or undifferentiatiated features. A higher mitotic index and areas of microvascular proliferation and/or necrosis can be observed although not prognostic. Immunophenotypically, midline tumors can express OLIG2, MAP2 and S100 with variable immunoreactivity for GFAP.

Diffuse hemispheric gliomas, H3-G34 mutant typically occur in older adolescents and young adults, with a reported median age of 25 in some adult cohorts (18). They can have histological features of glioblastomawith highly cellular, infiltrative astrocytic appearance and high mitotic activity. Microvascular proliferation and necrosis can be seen. Another pattern resembles embryonal tumors with small monomorphic hyperchromatic nuclei. These tumors may have GFAP positivity and typically loss of ATRX expression as well as nuclear p53 expression. OLIG2 is usually negative. HGGs in AYA patients that are H3-wildtype and IDH-wildtype represent a heterogeneous group of tumors and excluding adult-type GBM is an important part of the work up. As transformation from lower-grade tumors can also occur within this subtype, further investigations of molecular drivers, in particular B-Raf (BRAF) p.V600E, fibroblast growth factor receptor (FGFR), p53 and mutations in mismatch repair genes are also warranted.

In contrast to IDH-wildtype adult-type HGGs, the prognostic relevance of markers such as O^6^-methylguanine-DNA methyltransferase (MGMT) promoter methylation status in pediatric-type HGGs is unclear.

### Circumscribed astrocytic gliomas

Within this histologically heterogeneous group of tumors, pilocytic astrocytoma, high-grade astrocytoma with piloid features, pleomorphic xanthoastrocytoma, subependymal giant cell astrocytoma, and chordoid glioma, can be seen in the AYA population. Diagnosis currently relies heavily on histological characterization both for classification and grading. Relevant mutations, e.g. KIAA1549::BRAF fusion in pilocytic astrocytomas and BRAF p.V600E mutations in pleomorphic xanthoastrocytomas, need to be properly assessed in these tumors as molecular drivers can be both diagnostic (9) and define targeted therapy options.

### Clinical approach to gliomas in AYA and relevance of molecular testing

Upfront surgery for tissue diagnosis is recommended for most AYA gliomas unless serial MRIs reveal a stable, small, asymptomatic, non-enhancing tumor; or if a tumor is found in an eloquent, unresectable location. Clinical decision-making without tissue is warranted in some rare cases, e.g., optic pathway glioma in NF1 patients or diffuse intrinsic pontine glioma, but referral to specialist neurosurgeons at high-volume centers is recommended for AYA tumors in challenging locations. Maximal safe resection is recommended for all gliomas. Beyond the benefit of cytoreduction, adequate tissue is essential for morphologic assessment, immunohistochemical staining, and molecular testing.

#### High-grade gliomas

**Figure 1:**
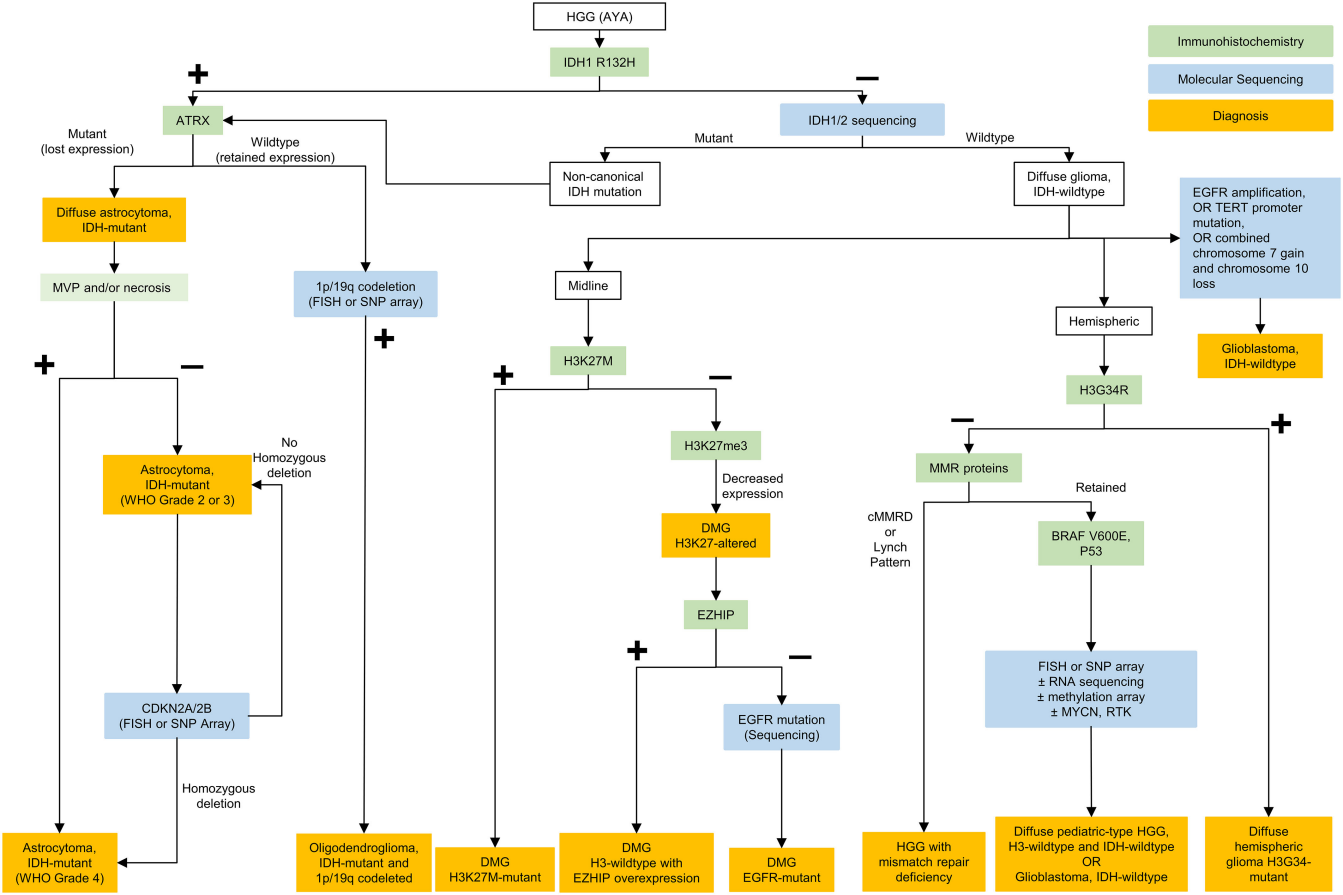
Testing algorithm for adolescent and young adult high grade gliomas. HGG, high grade gliomas; AYA, adolescent and young adults; IDH, isocitrate dehydrogenase; ATRX, ATRX chromatin remodeler; CDKN, cyclin-dependent kinase inhibitor; FISH, fluorescence *in situ* hybridization; SNP, single nucleotide polymorphism; WHO, World Health Organization; DMG, diffuse midline glioma; EGFR, epidermal growth factor receptor; RNA, ribonucleic acid; EZHIP, EZH Inhibitory Protein; MMR, mismatch repair; cMMRD, constitutional mismatch repair deficiency; BRAF, B-Raf; MYCN, N-myc proto oncogene; RTK, receptor tyrosine kinase; NTRK, neurotrophic tyrosine receptor kinase; TERT, telomerase reverse transcriptase.

Regardless of histological grading, it is recommended that AYA CNS diffuse gliomas be assessed for IDH-mutation through immunohistochemistry (IDH1 R132H antibody), and, if negative, genotyping for non-canonical IDH mutations. Clinically, the presence of an IDH mutation is prognostic, and should prompt further classification according to presence of 1p/19q codeletion (diagnostic of oligodendroglioma) and ATRX staining (with loss of staining diagnostic of astrocytic lineage). IDH sequencing to assess for non-canonical mutations in IDH1 or IDH2 is recommended in AYA tumors with negative staining for IDH1 R132H (**Figure 1**).

Currently, treatment of IDH-mutant tumors continues to rely on prognostic markers such as grade, performance status, age, extent of resection and neurological symptoms (14). About 25% of HGGs in AYAs are IDH-mutant and likely represent progression from a lower-grade glioma. In patients with high-risk IDH-mutant tumors (Grade 3 or 4, older age, residual tumor or symptomatic lesion), following initial resection, standard of care typically entails a combination of radiation followed by chemotherapy with either temozolomide or PCV (procarbazine, lomustine and vincristine) (14, 19). While this remains the standard of care for IDH-mutant glioma, there is increasing evidence that co-occurring mutations (20) or pathway alteration of PI3K, mTOR or AKT may be present (21), highlighting that testing for IDH mutations alone is insufficient in this group of diffuse gliomas. Mismatch repair deficiency (MMRD) has been associated in IDH-mutant gliomas following alkylating chemotherapy. These recurrent tumors tend to be of higher grade and less responsive to therapy, possible targeting of this resistance pathway is still under investigation and evaluating mutational burden and MMRD in these patients may help open clinical trial or therapeutic avenues. Hereditary MMRD in histologically high grade gliomas (constitutional MMRD and germline mutations in DNA mismatch repair genes such as *MLH1*, *MSH6* and *MSH2)* on the other hand has been described in pediatric and AYA patients and form a unique DNA methylation group (22). These tumors may benefit from approaches other than standard of care therapy including immune checkpoint inhibition (23). Therefore, testing AYA patients for MMRD (with immunostains, or sequencing) in newly diagnosed high grade IDH-mutant gliomas, or recurrent gliomas previously treated with alkylating chemotherapy is clinically valuable.

The approach to IDH-wildtype HGGs is different within the AYA cohort, where HGGs with molecular features of adult-type GBM are less frequently encountered than in older adults (24) (**Figure 1**). In midline IDH-wildtype HGG, alterations in H3 p.K27 should be assessed with H3 K27me3 and H3.3 p. K27M immunostaining. If there is a H3K27 alteration, molecular characterization may be helpful to allow enrollment in clinical trials. Assessment for a co-occurring mutation, although rare, may also be helpful to inform options for targeted therapy. Currently, H3 p.K27-altered diffuse midline gliomas (DMGs) carry a dismal prognosis in the pediatric population, similar to GBM in the adult population (median OS: 18.5 months) (24). Treatment of DMGs in AYA differs by institution, but radiation therapy is typically administered, and chemotherapy can be considered, although there is no established systemic therapy regimen within this population. Clinical trials outside of Canada are currently underway to test potential treatment strategies including with an oral small molecule imipridone dopamine receptor (DRD2) antagonist (ONC201, NCT02525692), a histone deacetylase inhibitor (panobinostat, NCT04804709) and with immunotherapeutics, including vaccines and cellular therapies (NCT04196413, NCT02960230), among other strategies.

In hemispheric HGGs, testing for H3 p.G34R with immunohistochemical staining is recommended. The presence of this mutation confers a better prognosis than adult-type GBM or H3 K27-altered DMG but a worse prognosis than IDH-mutant HGG (with median OS: 36.2 months) (24). Currently H3 G34R-mutant tumors are treated similarly to adult-type GBM, with maximal safe resection, concomitant chemoradiation, and typically adjuvant temozolomide. H3 p.G34R-mutant HGGs may also harbor activating platelet-derived growth factor receptor alpha (PDGFRA) mutations (25), which may have future treatment implications including with targeted agents such as imatinib.

In confirmed IDH- and H3-wildtype HGGs, further molecular sequencing can be conducted in a stepwise fashion in the absence of a readily available glioma-focused next-generation sequencing (NGS) panel. Ideally, molecular testing would be able to assess for relevant copy number alterations and chromosomal arm changes (gain of chromosome 7, loss of chromosome 10, *EGFR* amplification), along with other relevant mutations in “non-GBM” IDH-wildtype tumors (*TERT* promoter, *BRAFV600E, MYCN*, MMR*, EGFR, PDGFRA, p53*). Non-GBM IDH-wildtype gliomas, especially in AYA should be screened for other alterations with single nucleotide polymorphism [SNP] array and/or RNA sequencing panels when available.

In IDH-wildtype GBM, MGMT promoter methylation status is both a prognostic and predictive biomarker (26) and should be tested for. Although MGMT methylation status may be more impactful in determining the ideal regimen in elderly patients with GBM (27), it is often used in upfront clinical trials to determine eligibility and should ideally be done in all GBM patients regardless of age.GBM in AYA is treated with maximal safe resection, followed by concomitant chemoradiation and adjuvant temozolomide according to the Stupp protocol (28). Identification of molecular GBM is also essential for inclusion in clinical trials, which is encouraged for patients with both newly-diagnosed and recurrent GBM (29). While mutation-specific prognostic differences need to be better evaluated, as a group these pediatric type tumors often carry a better prognosis than GBM (5). In addition, identification of these mutations may open the door to targeted therapies with BRAF/MEK and FGFR inhibitors as well as clinical trial options. An algorithm for testing of HGGs in AYA patients is proposed in **Figure 1**.

#### Low-grade diffuse gliomas and circumscribed astrocytic gliomas

All gliomas typically undergo staining for IDH1 R132H, p53 and ATRX as a first step. Thereafter if ATRX is retained and p53 s negative, 1p/19q codeletion status is determined. If IDH1 R132H is negative, sequencing for non-canonical IDH is often pursued in young patients or if suggested by the clinical history. Lastly, in IDH-mutant astrocytomas, CDKN2A loss is evaluated for grading purposes (**Figure 1**). IDH-mutant LGGs in AYA tend to ultimately progress to HGG and treatment approaches may vary as discussed above.

**Figure 2:**
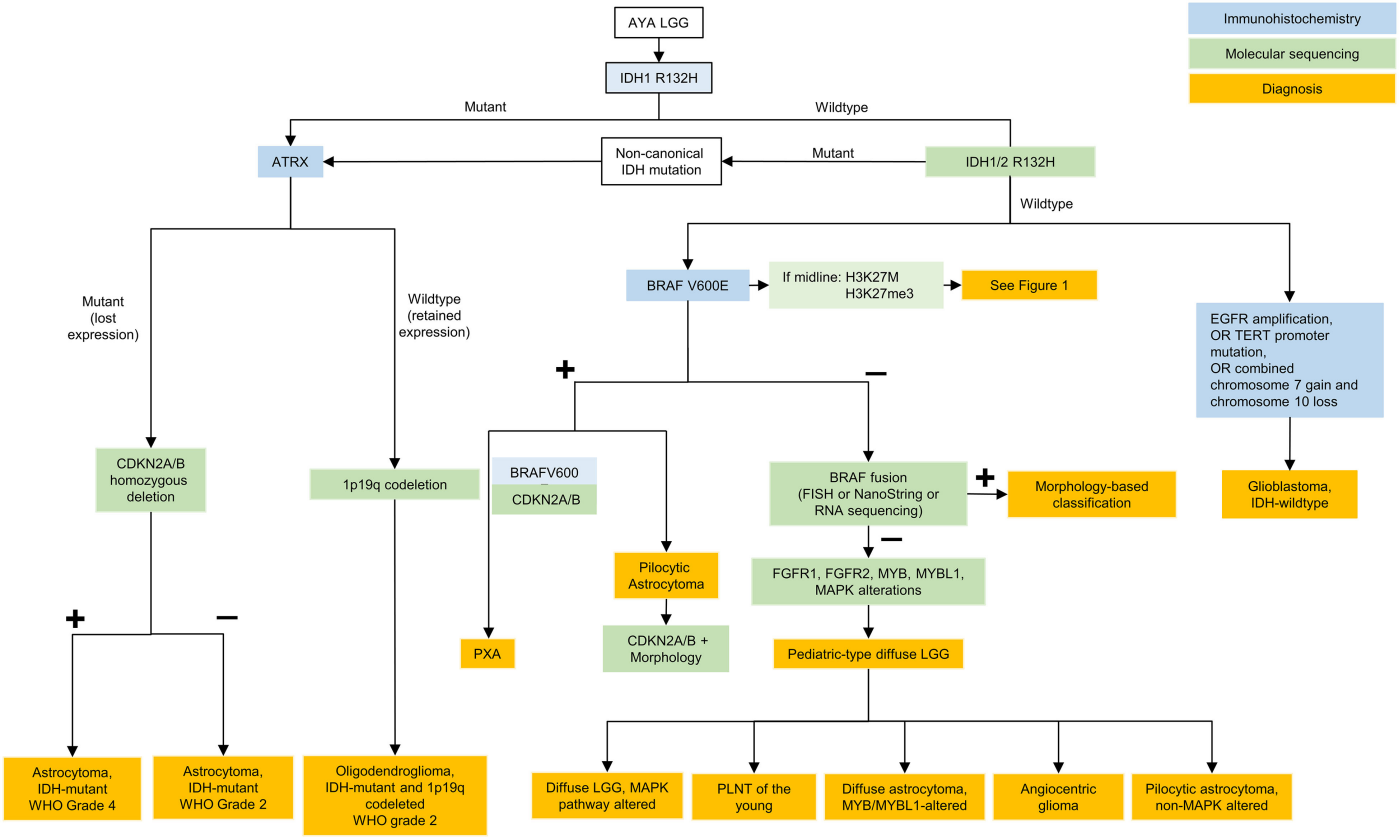
Testing algorithm for adolescent and young adult low grade gliomas. AYA, adolescent and young adults; LGG, low grade glioma; IDH, isocitrate dehydrogenase; ATRX, ATRX chromatin remodeler; CDKN, cyclin-dependent kinase inhibitor; WHO, World Health Organization; BRAF, B-Raf; FISH, fluorescence *in situ* hybridization; FGFR, fibroblast growth factor receptor; MYB, avian myelobalstosis viral oncogene; MYBL1, MYB Proto-Oncogene Like 1; MAPK, mitogen-activated protein kinase; PLGNT, pediatric low-grade neuroepithelial tumor; PXA, pleomorphic xanthoastrocytoma; EGFR, epidermal growth factor receptor; TERT, telomerase reverse transcriptase.

Importantly, if IDH negative, distinction must be made between molecular GBM and pediatric-type diffuse glioma. In this case, BRAF p.V600E may be tested using immunohistochemistry but other alterations require molecular diagnostics. Alterations in *FGFR*1, *FGFR2, MYB, MYBL1*, and BRAF can occur in AYA IDH-wildtype LGGs (**Figure 2**). Targeted treatments with FGFR or pan-RAF inhibitors represent viable approaches in the context of recurrent/residual disease that may decrease the need for other treatment modalities such as radiation therapy.

Circumscribed astrocytic gliomas are treated similarly to IDH-wildtype LGGs and often carry similar mutations in the RAS/MAPK pathway. In addition to these alterations, activating fusions of in neurotrophic tyrosine receptor kinase (NTRK) are exceptional but may offer options for targeted therapy with agents such as larotrectinib. For subependymal giant cell astrocytomas (SEGAs), mammalian target of rapamycin (mTOR) inhibitors (everolimus or sirolimus) can be given following maximal safe resection or in the context of recurrent disease.

### Glioneuronal tumors

The WHO CNS5 classification system defines several types of tumors with mixed neuronal and glial components. Gangliogliomas and dysembryoplastic neuroepithelial tumors (DNETs) are the two most common glioneuronal tumors. Other glioneuronal tumors include diffuse glioneuronal tumor with oligodendroglioma-like features and nuclear clusters (DGONC), rosette-forming glioneuronal tumors (RGNT), papillary glioneuronal tumors, myxoid glioneuronal tumors, diffuse leptomeningeal glioneuronal tumors (DLGNT), gangliocytomas, multinodular and vacuolating neuronal tumors (MVNTs), and Lhermitte–Duclos disease. Central neurocytomas are categorized as neuronal tumors.

Glioneuronal tumors are uncommon, though they make up a large portion of long-term epilepsy-associated tumors (30). In Canada, the age-standardized annual incidence rate of glioneuronal tumor diagnosis among AYA patients is 0.42 per 100,000 (compared to 0.33 and 0.22 per 100,000 in younger and older patients, respectively) (12).

### Clinical approach to glioneuronal tumors in AYA and relevance of molecular testing

Surgical resection is the most important treatment for symptomatic, circumscribed glioneuronal tumors which can also result in effective and durable anti-seizure control. For recurrent or widespread disease, other treatment options include radiotherapy or chemotherapy. Glioneuronal tumors occasionally harbor potentially targetable molecular alterations (**Table 1**). In DNETs, germline or somatic FGFR1 mutations are common, while BRAF p.V600E mutations are rare (31). In gangliogliomas, BRAF p.V600E mutations occur in 10–60% of cases (depending on the tumor location). Rosette-forming glioneuronal tumors can have FGFR1 mutations with co−mutation of PIK3CA and NF1 (32). Targeted therapies are currently under investigation for the treatment of glioneuronal tumors.

## Ependymoma

**Figure 3:**
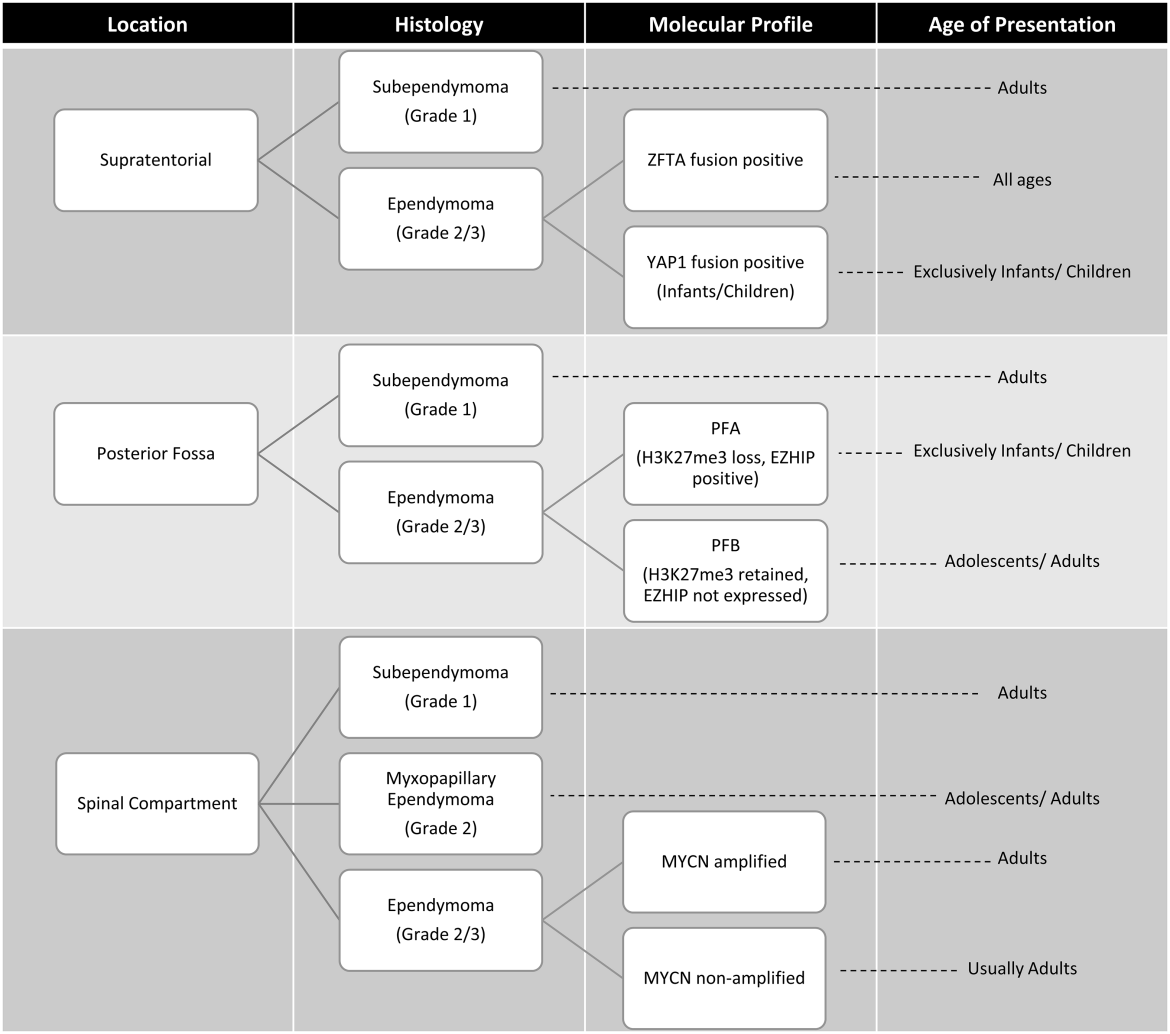
Age at presentation and classification based on anatomic site, histology and molecular features of ependymomas (33, 34). ZFTA, zinc finger translocation associated; YAP1, yes-associated protein 1; posterior fossa type A (PFA) and posterior fossa type B (PFB) MYCN, N-myc proto oncogene.

**Figure 4:**
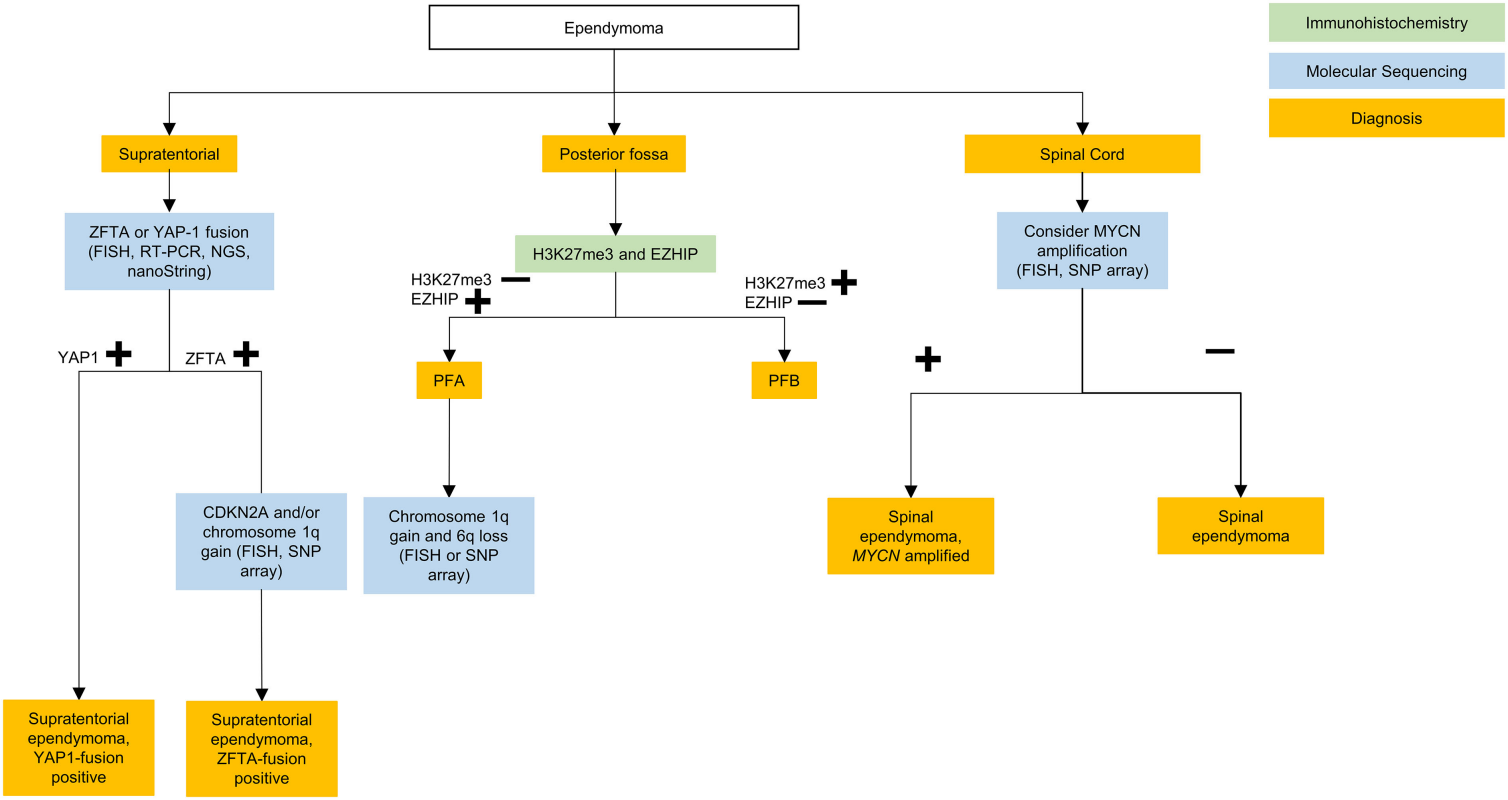
Testing algorithm for adolescent and young adult ependymomas. ZFTA, zinc finger translocation associated; YAP1, yes-associated protein 1; FISH, fluorescence *in situ* hybridization; RT-PCR, reverse transcriptase polymerase chain reaction; NGS, next generation sequencing; PFA, posterior fossa type **(A)** PFB, posterior fossa type **(B)** MYCN, N-myc proto oncogene; CDKN, cyclin-dependent kinase inhibitor; SNP, single nucleotide polymorphism.

Since the 2016 iteration of the WHO classification of CNS tumors, advances in the understanding of the molecular characteristics and biology of ependymomas has prompted a revised classification. In WHO CNS5, ependymomas continue to be grouped based on anatomical site across the supratentorial, posterior fossa and spinal compartments (1), with enhanced focus on molecular features. Prior to the WHO CNS5 revisions, methylome profiling and genomic studies revealed at least 9 molecular subgroups of ependymoma that were superior in risk stratification compared to histopathologic classification (33). The 5th edition now classifies ependymomas into 10 subgroups based on a combination of histopathological, anatomical and molecular features (**Figure 3** **).** Ependymomas within each compartment can be designated either grade 2 or 3 depending on histologic features, as data regarding grading of molecularly defined subtypes is still immature (8). Within WHO CNS5, the term “anaplastic” has been removed, and the morphological variants of classical ependymoma (papillary, clear cell, and tanycytic) are no longer recognized as ependymoma subtypes due to lack of clinical utility, and are instead included as histological patterns (8). A simplified algorithm for classification is presented in **Figure 4**.

### Clinical approach to ependymoma in AYA and relevance of molecular testing

Treatment stratification in ependymoma is currently based on anatomical location, extent of resection, grade and the presence of dissemination rather than molecular subtype (9, 35, 36). Therefore, the standard of care, beginning with maximal resection with consideration of post-operative conformal radiation, has not yet changed based on the molecularly expanded diagnostic schema (37). However, current clinical consensus suggests that pending more trial data, treatment should be tailored to the distinct molecular variants of ependymoma (38).

Upfront adjuvant chemotherapy has not shown benefit in adult ependymoma regardless of grade or subtype, but temozolomide and lapatinib can be considered in the recurrent setting based on phase II data (39, 40). There is no established role for upfront chemotherapy in the management of pediatric ependymoma (41), however preliminary results from the ACNS0831 trial shows some benefit to adjuvant maintenance chemotherapy in gross total or near totally resected, newly diagnosed ependymoma in children (42). Other therapies for recurrent ependymoma include re-operation (43) – ideally a gross total resection for locally-recurrent disease – and re-irradiation (43, 44).

Although the advances in molecular diagnosis have not yet changed the standard of care in ependymoma, molecular subgrouping of ependymoma allows for more precise prognosis and tailored treatment intensity (35). Clinical trials can now better stratify and compare novel investigational treatments specific to the 10 ependymoma subtypes in the WHO CNS5. Molecular diagnosis is essential to the development of new targeted treatments in this rare group of tumors.

#### Supratentorial ependymoma

The WHO CNS5 classification now lists two molecular groups of STE, characterized by their recurrent genetic alterations. Most relevant to the AYA population is defined by zinc finger translocation-associated (*ZFTA*; previously C11orf95) gene fusions. This is the same as the reticuloendotheliosis viral oncogene homolog A (RELA) fusion-positive ependymoma group introduced in the 4^th^ edition of the WHO CNS guidelines, with the name changed prompted by the slightly increased prevalence of the ZFTA gene as a fusion partner (9). The second type is defined by yes-associated protein 1 (*YAP1*) gene fusions which is largely restricted to infants and young children (33).

Suggested molecular work-up of STE in AYA patients involves testing for *ZFTA* fusions, which can be identified *via* fluorescence *in situ* hybridization (FISH), reverse transcriptase polymerase chain reaction (RT-PCR), next generation sequencing (NGS), or nanoString (45). In the AYA population, if a *ZFTA* fusion is not present then alternate diagnoses such as GBM with ependymal differentiation (46), or MN1-fused or BCOR-fused neuroepithelial tumors, should be considered (47, 48).

Following gross total resection, observation may be considered for grade 2 *ZFTA* fusion STE (49). Additional FISH, SNP array, or methylation profiling for homozygous *CDKN2A* deletion can be subsequently performed for *ZFTA* fusion STE, as it is a prognostic marker for increased risk of local and distant disease progression (50–53). However, further trials are needed prior to the recommendation of molecularly tailored treatment for STE (38).

#### Posterior fossa ependymoma

Posterior fossa ependymomas lack recurrent mutations, and are instead classified based on epigenetic methylation profiling (54). In WHO CNS5 they are categorized into two main groups based on global levels of histone H3 K27 trimethylation, with group PFA exhibiting loss of trimethylation and over-expression of EZH inhibitory protein (EZHIP), and PFB exhibiting trimethylation retention (55). Most posterior fossa ependymoma occurring in adults are PFB while the great majority of PFA ependymoma occur in children younger than 8 years of age, with a median age of diagnosis of 3 years (33). It should be noted that while retention of H3K27me3 is characteristic of PFB, it is by no means specific and if the diagnosis is in doubt, then methylation profiling may be of help here.

In the AYA population, PFB is more common and carries a favorable prognosis compared to the PFA subgroup (33, 55–57). In a large retrospective multicohort of patients with ependymoma, PFA occurred in 10% of adults with posterior fossa ependymoma and within this group outcome was not affected by age. PFA occurred equally between the ages of 18-54 years (56). PFA versus PFB can be differentiated, as mentioned above, on immunohistochemistry (IHC) with H3K27me3 and EZH inhibitory protein (EZHIP) staining. Methylation profiling may also be used (55).

PFB ependymoma should be treated with gross total resection following which the prognosis is excellent however trials are required to further ascertain if these patients can be observed as re-resection and radiation therapy at recurrence may represent a viable option (38, 56). Patients with PFA should also be treated with gross total resection, when possible, as subtotal resection carries a high risk of poor outcome. Unfortunately, due to the frequent involvement of the brainstem, there is often residual disease, and the recommendation is therefore for adjuvant therapy in the form of external-beam irradiation, and hopefully novel therapies in the future.

#### Spinal compartment ependymoma

Spinal cord ependymomas typically occur in young and middle aged adults (39). Among these, the WHO CNS5 now grades myxopapillary ependymomas as grade 2 given comparable clinical outcomes to classic spinal cord ependymomas (58). WHO CNS5 also recognizes a new distinct molecular subtype of rare spinal cord ependymoma characterized by *MYCN* amplification, that is associated with older age, early dissemination and poor prognosis (59, 60).

Spinal and myxopapillary ependymoma are defined morphologically rather than molecularly (8), and therefore molecular testing is not necessary unless the tumor appears aggressive or disseminated, in which case *MYCN* amplification should be tested *via* FISH or SNP arrays (58, 59).

Spinal ependymomas are treated like other ependymomas, with maximal resection followed by observation or radiation depending on extent of resection, grade and evidence of dissemination (36). The treatment approach to myxopapillary ependymoma is en bloc gross total resection and, provided the capsule is not violated, adjuvant radiation is not required. In the case of capsule violation or subtotal resection, then adjuvant radiation should follow (36).

#### Subependymoma

Additionally, within each anatomic site, subependymomas can occur. These are identified histopathologically and do not require molecular testing (8). These grade 1 tumors are most commonly diagnosed in adults over the median age of 40 years, and therefore do not often present in the AYA age range and their management will not be further discussed (33).

## Medulloblastoma and embryonal tumors

Embryonal tumors of the CNS are highly malignant and poorly differentiated tumors of neuroepithelial origin. Of these, medulloblastoma is one of the most common malignant brain tumors in children, however in adults these tumors are less common, accounting for less than 1% of all intracranial malignancies in adults (61). While most cases of medulloblastomas are diagnosed in children prior to the age of 15 years (median age: 9 years) it is the most common malignant embryonal tumor in the AYA population. As medulloblastoma arising in childhood vs adulthood have different subgroup enrichment and prognostic markers, treatment strategies and outcome predictors should also be differentiated. However, clinical trials are limited; standardized treatment are lacking in the adult population and decisions are often extrapolated from available pediatric standards of care.

The WHO CNS5 classification retains the four main molecular subgroup and morphologic classification from the 2016 classification of medulloblastoma. The molecular groups are: Wingless-type (WNT)-activated; Sonic Hedgehog (SHH)-activated (*TP53* wildtype and mutated); group 3 and group 4 (non-WNT/non-SHH). The morphologic groups are classic; desmoplastic/nodular; and anaplastic/large cell and medulloblastoma with extensive nodularity (MBEN) – with the expectation that these are reported in a layered diagnostic fashion together with other molecular prognostic features. Additional sub-subgrouping with methylation array is also discussed but the clinical utility of these, particularly in the AYA age group, is not clear at present.

Tumor location tends to be subgroup associated and is thought to be related to cell of origin, with WNT tumors found in the cerebellopontine angle cistern and cerebellar peduncle, SHH-activated tumors arising in the cerebellar hemispheres, and Group 3 or 4 tumors occurring within the midline and fourth ventricle (62, 63). In children, WNT medulloblastoma can also be found in the Foramen Luschka and fourth ventricle (64).

Within the four main molecular subgroups, adult medulloblastoma differs from their pediatric counterparts (65). A majority (60%) of adult medulloblastomas fall within the SHH group, followed by Group 4 and WNT tumors; Group 3 tumors are rare in adults. Within each subgroup further transcriptional and epigenetic changes have shown to contribute to risk stratification (66–68). An overview of the subtypes of medulloblastoma is presented in **Table 2**.

**Table 2:** Medulloblastoma subgroups with relevant clinical, molecular information and risk stratification.

Subgroup	WNT-activated	SHH-activated (TP53 wildtype and mutated)	Group 3	Group 4(non-WNT, non-SHH)
***Clinical Information***				
Age at diagnosis	Child > 4 years old, adolescents and adults	Bimodal, most often occurring in infants and adults	Infants and young children	Childhood and adolescents
Anatomic location	Midline with involvement of brainstem or in cerebellar peduncle and cerebellopontine angle cistern	Cerebellar hemispheres	Midline vermian location adjacent to 4th ventricle	Midline vermian
Histology	Classic, rarely LCA	Desmoplastic/nodular, Classic, LCA	Classic, LCA	Classic, LCA
***Molecular characteristics***				
Recurrent gene amplifications		MYCN* GLI1 or GLI2*	MYC* MYCN* OTX2	SNCAIP MYCN* OTX2 CDK6
Recurrent single-nucleotide variants/mutations	CTNNB1 DDX3X SMARCA4 TP53 CSNK2B	PTCH1 TERT** SUFU* SMO** TP53 U1 snRNA** DDX3X**	SMARCA4 KBTBD4 CTDNEP1 KMT2D	KDM6A SMYM3 KTM2C KBTBD4
Cytogenetic events	Loss of chromosome 6*	Gain of chromosome 3q or 9p Loss of chromosome 3p, 9q, 10q, 14q, 17p	Gain of chromosome 1q Loss of chromosome 8, 10q,11, 16q Isochromosome 17q	Gain of chromosome 7, 18q Loss of chromosome 8, 11, 13q Isochromosome 17q
***Outcome Predictors***				
Childhood	nuclear B-catenin accumulation#	TP53 mutations##	MYC amplification##	Chromosome 8 loss# Amplification of MYC or MYCN ##
Adult		10q, 3p, or 17p loss## PTCH1 mutations## TP53 mutations##	Isochromosome 17q##	Chromosome 8 loss # CDK6## Isochromosome 17q##

LCA, Large cell anaplastic; *more likely to be associated with childhood medulloblastoma, **more likely to be associated with adult medulloblastoma, # associated with better prognosis, ## associated with inferior prognosis.

WNT tumors are found in both children and adults; 15-20% of adult medulloblastomas are of WNT subgroup and, in contrast to pediatric cases, are less likely to harbor monosomy 6. Those diagnosed in childhood (prior to age 16) have excellent prognosis, with 10-year event free survival >95% (69, 70). Most WNT medulloblastomas have mutations in exon 3 of the *CTNNB1* gene which results in reduced cytoplasmic degradation and nuclear accumulation of B-catenin, a transcription factor coactivator. In children, nuclear B-catenin accumulation is associated with excellent prognosis whereas this prognostic value has not been shown in adults (71); this difference may be due to differing treatment regimens rather than to intrinsic biological differences.

In adults, the most frequent group of medulloblastoma is SHH-activated, and the most common subset of SHH-activated medulloblastomas are *TP53* wildtype, accounting for up to 70% of cases (69, 72). In contrast to the pediatric population, a P53 mutation is less likely to be associated with hereditary cancer predisposition, specifically Li-Fraumeni syndrome, and is not as negative a prognostic marker in adults. They tend to have an enrichment for *TERT* promoter mutations, demonstrate loss of function mutations or deletions in *PTCH1*, or copy number changes, specifically 10q loss (73–77). Adult SHH-medulloblastoma have frequent upstream pathway alterations (*PTCH1* and *SMO* mutations), but infrequent downstream alterations (*SUFU, MYCN* amplifications) (73, 78). In a group of older children and adolescents with SHH medulloblastoma, germline or somatic *TP53* mutations were associated with poor outcomes (72). In adults, germline *TP53* mutations are rare or non-existent (77). Furthermore, the presence of 10q loss serves as a strong predictor for poor survival specifically (68, 71, 77), and SHH tumors with chromosome 3p loss, 17p loss and *PTCH1* mutations have inferior outcomes.

Group 3 medulloblastoma are primarily seen in infants and older children. They tend to be male patients and frequently present with disseminated disease with a poor prognosis, especially in the context of *MYC* amplification (79). Other cytogenetic features include isochromosome 17q and chain of chromosome 8q (80). Gene mutations are infrequent, but tend to include *SMARCA4, KBTBD4, CTDNEPI* and *KMT2D* (80–82).

Group 4 medulloblastoma frequently have cytogenetic aberrations with gain of chromosome 7 or 17q and deletions of chromosome 8, 11 or 17p, or isochromosome 17q (80). Chromosome 8 loss has been shown in both pediatric and adults to be associated with increased survival, whereas other pediatric markers such as whole chromosome 11 loss have not been found to be prognostic in adults (77). Pediatric Group 4 medulloblastoma with chromosome 8 loss has been associated with a survival advantage (83). This has also been recently shown to be true in adults (77). Amplifications in *MYCN* and *CDK6* are seen, as is overexpression of *PRDM6* and mutations in histone modifying genes*, KDM6A, AMYM3, KMT2C* and *KBTBD4* (79, 80). Amplification of *MYC* or *MYCN* has been shown to be associated with poor survival in pediatric medulloblastoma, but are rare in adults (71). In contrast, *CDK6* is almost exclusively found in adults and correlated with adverse outcomes (71).

### Clinical approach to medulloblastoma in AYA and relevance of molecular testing

Like gliomas and ependymomas, standard of care management for medulloblastomas begins with maximal surgical resection for cytoreduction, as well as histopathologic and molecular diagnosis. The historical Chang staging criteria (M0–M4) are still used for medulloblastoma. M0 represents no evidence of metastatic disease; M1 is those with positive cerebrospinal fluid (CSF) cytology without gross visible tumor radiographically; M2 is intracranial metastasis; M3 is overt metastasis within the spinal subarachnoid space; and M4 is disease outside of the neuraxis. Extraneural metastases, especially late metastases, have been reported in adult cases and most commonly involve bone, and rarely lymph node, visceral organs and bone marrow (84–87). M-stage at diagnosis is prognostic in children, but its role in adults is less clear (88–91). Patients tend to be classified as average or high risk based on presence of metastatic disease and extent of resection. Average risk patients are those with 1.5cm^2^ or less residual disease, no metastatic disease on MRI brain and spine, and absence of malignant cells in the CSF *via* lumbar puncture, whereas those with residual disease or presence of metastatic disease on imaging or CSF are classified as high risk. In children extent of surgical resection, specifically gross total resection with 1.5cm^2^ or less residual disease has been shown to be prognostic. However, in adults, the prognostic value of complete resection is less clear. Nonetheless guidelines recommend a gross total resection if possible (92–94).

Diagnostic classification of medulloblastoma into the 4 main subgroups is accomplished with IHC and molecular methods, with the latter being preferred. IHC testing of beta-catenin can identify tumors in the WNT subgroup (beta-catenin positive), while the SHH subgroup, group 3, and group 4 are negative for beta-catenin. It is important to note that beta-catenin IHC may not entirely be reliable and should be interpreted with caution; instead, molecular subgrouping is preferred. GAB1 and filamin-A are positive *via* IHC in the SHH subgroup, but negative in the group 3 and 4 subgroups. There is no reliable immunohistochemical method for distinguishing groups 3 and 4.

Medulloblastoma subgrouping is most reliably performed by molecular methods, including nanoString assay, or methylation profiling. Additional copy number alterations of potential prognostic significance maybe be obtained from the methylation array or *via* SNP arrays or FISH, but are not always necessary. Such molecular subclassification has become routine for the diagnosis of pediatric medulloblastoma, whereas the clinical benefit in adults is less clear (92).

Within Canada, the standard treatment approach for childhood medulloblastoma (> 3–6 years of age) and average risk medulloblastoma is treatment as per the ACNS0331 protocol (95, 96). This involves upfront maximal safe surgical resection followed by craniospinal irradiation (CSI) (23.4 Gy) with a boost to the tumor bed, with a total dose of 54–55.8 Gy, with concomitant vincristine. This is then followed by 9 cycles of multi-agent chemotherapy including vincristine, CCNU, cisplatin and cyclophosphamide. Comparatively, those with high risk medulloblastoma are treated per the ACNS0332 protocol, Regimen A (95, 96), with 36 Gy CSI with a boost to the tumor bed/posterior fossa to a total of 54–55.8 Gy followed by 6 cycles of chemotherapy with vincristine, cisplatin, and cyclophosphamide.

As medulloblastoma is infrequent in adults, treatment protocols are quite heterogeneous and more intense systemic protocols have reported subsequent toxicities in adult patients (96, 97). Most published cohorts of adult medulloblastoma patents are limited to retrospective studies and randomized trials are lacking. Current adult approaches include maximal safe resection followed by craniospinal irradiation with or without adjuvant chemotherapy irrespective of risk category (92). Treatment recommendations are generally derived from pediatric trials, retrospective analysis of adult cohorts within pediatric trials, and prospective single-arm trials in adults (92, 94, 98–101). Although craniospinal radiation has been shown to be necessary, there is controversy regarding the appropriate dose of radiation in adults, with most opting for 36 Gy to the neuraxis and localized dose escalation to the tumor bed. Reduced dose CSI to 23.4 Gy in combination with chemotherapy has been used in pediatric trials (102) and is being investigated in adults (NCT01857453). Furthermore, there are ongoing discussions regarding appropriate radiation dosing to the tumor bed in adult patients, with doses higher than 50 Gy associated with an improved outcome (97).

Most retrospective studies in adults suggest that adjuvant chemotherapy is associated with improved survival relative to craniospinal radiotherapy alone in both average and high-risk individuals (98, 103), however the added value to high-risk adults is less established than in children. The typical chemotherapy regimen used in adults is the Packer regimen (adjuvant vincristine, cisplatin, and lomustine up to 8 cycles) (102). Weekly vincristine during RT is often omitted or dose modified in adults due to increased risk of toxicity. Overall, tolerance to the Packer regimen is worse in adolescents and adults compared to children (104), with dose modifications or early termination required by cycle 4 in nearly 60% of patients (99). Therefore, identifying therapeutic targets utilizing molecular characterization may improve patient care and should aim to reduce toxicities associated with therapy. An example of this is the SHH pathway as a potential candidate for targeted therapy for patients for recurrent or refractory disease. Some early clinical trials have shown efficacy of the SMO inhibitor vismodegib in recurrent SHH medulloblastoma phase I trials. The majority of pediatric relapsed cases had P53 mutant SHH and downstream activation such as SUFU mutations limiting vismodegib’s effectiveness, but these alterations are uncommon in adults. Thus, in theory adult SHH-activated medulloblastoma patients may benefit from vismodegib with improved response rates noted in almost half of patients when used in the recurrent setting as demonstrated in phase II trials. This response may, however, be short lived, as PFS was consistently less than 4 months (105, 106). Recently, a phase I/II study evaluating vismodegib and temozolomide vs. temozolomide alone in recurrent/refractory medulloblastoma in adults showed no added toxicity, but failed to show improvement in PFS (107). Further studies are required to elucidate the role of vismodegib and other potential targeted therapies for medulloblastoma.

Pediatric SHH medulloblastoma patients should undergo genetic counselling for evaluation of germline *TP53* and SHH pathway mutations (Gorlin syndrome) and WNT patients without somatic CTNNB1 mutations require genetic counselling for APC sequencing. In contrast, adult SHH patients do not routinely require referral for genetic testing given the rarity of germline *TP53* mutations in older patients, unless there are other clinical or familial concerns.

## Discussion

AYA patients represent a unique group of patients whose care spans both pediatric and adult treatment centers. As a result, the diagnostic and treatment approaches to their biologically unique tumors have been heterogeneous across Canada. The incorporation of diagnostic and prognostic molecular biomarkers, culminating in the WHO CNS5, has led to a rapid evolution of our understanding of CNS tumors in AYA patients. WHO CNS5, in particular, highlights the distinct pediatric and adult subtypes within gliomas, and the need for separation of these entities for treatment considerations and prognostication. New classifications are especially useful if each element is prognostically distinct or if each differentially responds to therapies. In particular, molecular classification of CNS tumors can assist with accurate prognostication, can open up clinical trial options, and can occasionally allow for targeted therapy or treatment de-escalation. Looking to the future, access to molecular sequencing, combined with liquid biopsies could also present a unique chance to refine less invasive diagnostic and prognostic biomarkers in AYA patients (108).

While this review provides some recommendations for testing, national implementation within the Canadian single-payer system remains challenging, because provincial insurance coverage often dictates access to advanced molecular testing. Because AYA CNS tumors are relatively rare, centralized testing may provide the only feasible option to better identify and molecularly characterize these tumors to ensure suitable therapy, and follow-up, is provided to this group of patients. In larger Canadian provinces, streamlining testing at one or two major centers could ensure best use of resources and allow for better development and validation of next generation sequencing panels. However, in less populous provinces, inter-provincial collaborations may be required for timely access to testing. The challenges of a single-payer system may ultimately ensure that technological advances that translate into improved patient care are implemented more uniformly and equitably, although this process may be slower in its development.

In this vulnerable patient population, where treatment strategies can have long term sequelae, it is particularly important that research and clinical efforts recognize not only the unique biology but also the unique effects of age on the ability to endure or respond to treatments. Clinical trial designs will need to evolve to address these challenges. For example, current trials that incorporate AYA as a group (and do not segregate pediatric vs adult patients) remain scarce but are essential. In addition, sample size calculations and trial designs must take into account the rarity of these tumors, and accrual will often have to depend on multi-institutional and international collaborations. Cooperative group studies that span the 18-21 age group, such as ARST1321 trial of non-rhabdomyosarcoma soft tissue sarcomas that was developed by both NRG Oncology and the Children’s Oncology Group, are able to successfully accrue participants aged <18 and >18 (109). Bridges and platforms for communication need to be built between adult and pediatric neuro-oncologists, radiation oncologists, neurosurgeons, and neuropathologists to improve care in this population, and to ensure that care is consistent across the country. Similar collaborations between Canadian research organizations, such as the Canadian Clinical Trials Group, Canadian Cancer Clinical Trials Network (3CTN) and C17 Council are necessary to develop high-impact, clinically relevant studies spanning the pediatric, AYA and adult disease spectrum. As the need for better molecularly guided therapies and interdisciplinary and interprovincial collaborations has been recognized, efforts initiated by our Canadian Adolescent and Young Adult Brain Tumour Consortium has led to the creation of initiatives such as a pan-Canadian multidisciplinary molecular tumor boards to help address these gaps. This current work reflects our goal to provide a unified, resource-effective, clinically oriented approach to molecular testing in light of the new WHO CNS5 classification, that will help ensure that all AYA patients are eventually cared for within this evidence and precision-medicine based framework. Lastly, while the molecular testing gap is closing, access to targeted therapies for AYA continues to lack behind and should be the focus of future advocacy efforts.

While our collaborative efforts are still in their infancy, improved identification and classification of these tumors for Canadian AYA brain tumor patients will help inform standards of practice and help accelerate research efforts to provide more precise therapies and may spare long term side effects of some of the current treatment strategies in a population that needs it the most.

## Author contributions

All authors listed have made a substantial, direct, and intellectual contribution to the work and approved it for publication.

## Acknowledgments

The authors would like to thank Ms. Aimee Chan for her editorial assistance

## Conflict of interest

SY is a member of advisory board of Amgen, AstraZeneca, Bayer, Incyte, and Roche.

Sunit Das receives laboratory research funding from Alkermes. He is a speaker for the Congress of Neurological Surgeons and American Association of Neurological Surgeons. He is a member of the advisory board of the Subcortical Surgery Group and Xpan Medical. He serves as the Provincial Lead for CNS Tumours at Ontario Health (Cancer Care Ontario).

The remaining authors declare that the research was conducted in the absence of any commercial or financial relationships that could be construed as a potential conflict of interest.

## Publisher’s note

All claims expressed in this article are solely those of the authors and do not necessarily represent those of their affiliated organizations, or those of the publisher, the editors and the reviewers. Any product that may be evaluated in this article, or claim that may be made by its manufacturer, is not guaranteed or endorsed by the publisher.
